# Induction of CD36 and Thrombospondin-1 in Macrophages by Hypoxia-Inducible Factor 1 and Its Relevance in the Inflammatory Process

**DOI:** 10.1371/journal.pone.0048535

**Published:** 2012-10-31

**Authors:** Dolores Ortiz-Masià, Irene Díez, Sara Calatayud, Carlos Hernández, Jesús Cosín-Roger, Joaquín Hinojosa, Juan V. Esplugues, María D. Barrachina

**Affiliations:** 1 Departamento de Farmacología and CIBERehd, Facultad de Medicina, Universidad de Valencia, Valencia, Spain; 2 FISABIO, Hospital Dr. Peset, Valencia, Spain; 3 Hospital de Manises, Valencia, Spain; University of Colorado Denver, United States of America

## Abstract

Inflammation is part of a complex biological response of vascular tissue to pathogens or damaged cells. First inflammatory cells attempt to remove the injurious stimuli and this is followed by a healing process mediated principally by phagocytosis of senescent cells. Hypoxia and p38-MAPK are associated with inflammation, and hypoxia inducible factor 1 (HIF-1) has been detected in inflamed tissues. We aimed to analyse the role of p38-MAPK and HIF-1 in the transcriptional regulation of CD36, a class B scavenger receptor, and its ligand thrombospondin (TSP-1) in macrophages and to evaluate the involvement of this pathway in phagocytosis of apoptotic neutrophils. We have also assessed HIF-1α, p38-MAPK and CD36 immunostaining in the mucosa of patients with inflammatory bowel disease. Results show that hypoxia increases neutrophil phagocytosis by macrophages and induces the expression of CD36 and TSP-1. Addition of a p38-MAPK inhibitor significantly reduced the increase in CD36 and TSP-1 expression provoked by hypoxia and decreased HIF-1α stabilization in macrophages. Transient transfection of macrophages with a *miHIF-1α*-targeting vector blocked the increase in mRNA expression of *CD36* and *TSP-1* during hypoxia and reduced phagocytosis, thus highlighting a role for the transcriptional activity of HIF-1. CD36 and TSP-1 were necessary for the phagocytosis of neutrophils induced by hypoxic macrophages, since functional blockade of these proteins undermined this process. Immunohistochemical studies revealed CD36, HIF-1α and p38-MAPK expression in the mucosa of patients with inflammatory bowel disease. A positive and significant correlation between HIF-1α and CD36 expression and CD36 and p38-MAPK expression was observed in cells of the lamina propria of the damaged mucosa. Our results demonstrate a HIF-1-dependent up-regulation of CD36 and TSP-1 that mediates the increased phagocytosis of neutrophils by macrophages during hypoxia. Moreover, they suggest that CD36 expression in the damaged mucosa of patients with inflammatory bowel disease depends on p38-MAPK and HIF-1 activity.

## Introduction

Inflammation is part of the complex biological response of vascular tissues to harmful stimuli such as pathogens or damaged cells, by which the injurious stimuli should be removed and the healing process initiated. Hypoxia and p38 group mitogen-activated protein kinase (p38-MAPK) have been associated with inflammatory diseases [1]–[3]. Hypoxia inducible factor-1 (HIF-1) is the main regulator of the transcriptional response to hypoxia and its activity is modulated by the p38-MAPK signaling pathway [4], [5]. Stabilized HIF-1α is observed in several inflamed tissues and, in the case of clinical colitis it has been detected in epithelial and inflammatory cells [6]–[8]. In epithelial cells HIF-1 induces the expression of genes involved in mucosal defence and repair, such as mucin 3 and trefoil factors [9], [10], and has been identified as a critical factor for barrier protection. The role it plays in inflammatory cells seems to be more complex, with specific functions being reported according to the type of cell. HIF-1 increases the expression of ß_2_ integrin, which promotes neutrophil binding to the endothelium [11], [12], and activation of HIF has been reported during macrophage differentiation [13]. At the inflammatory focus, HIF-1 prevents the apoptosis of neutrophils and mediated bacterial phagocytosis by macrophages [12], [14]–[16]. Considered as a whole, these observations demonstrate a protective effect of HIF-1 in epithelial cells and point to a key role in the activation of the innate immune response against pathogens and injury. However, the involvement of HIF-1 and its transcriptional activity in the clearance of cellular debris and apoptotic cells mediated by macrophages [17], a crucial process in the resolution of inflammation, is yet to be clarified.

CD36 is a heavily glycosylated transmembrane protein belonging to an evolutionarily conserved family of scavenger receptors. This multifunctional receptor is expressed on the surface of different cells, including macrophages, and is known to be involved in scavenger recognition of apoptotic cells [18], [19], exogenous pathogens and their inflammatory compounds [20]. The interaction between CD36 and apoptotic cells seems to be mediated specifically by thrombospondin-1 (TSP-1), an extracellular matrix glycoprotein that bridges apoptotic cells, CD36 and the vitronectin receptor, thus creating a phagocytically active ternary complex [21]. CD36 expression is transcriptionally controlled by the nuclear receptor PPARγ [22]. However, a recent study has demonstrated that inflammatory macrophages, in which activation of PPARγ is down-regulated, are endowed with an alternative mechanism of CD36 expression [23]. Given that HIF-1 has been related to CD36 expression in vascular and smooth muscle cells [24] but that little is known about the regulation of CD36 and TSP-1 in hypoxic macrophages, we set out to analyse the role of HIF-1 and its transcriptional activity in phagocytosis of neutrophils. Our results show a HIF-1 dependent induction of CD36 and TSP-1 in macrophages which regulates hypoxia-induced phagocytosis of apoptotic neutrophils. They also suggest that CD36 regulation by HIF-1is implicated in the damaged mucosa of patients with inflammatory bowel disease.

## Materials and Methods

### Ethics Statements

All protocols were approved by the Ethics Committee of the Faculty of Medicine, University of Valencia. The experiments performed with human samples were approved by the Institutional Review Board of Manises’ Hospital (Valencia). Written informed consent was obtained from all patients.

### Cell Culture and Treatment

Human monocytes (U937 and THP1, European Collection of Cell Culture Salisbury, UK) were cultured in RPMI medium (Sigma Chemical CO, St. Louis, MO) with 10% inactivated bovine fetal serum (FBS, Lonza, Basel, Switzerland), 1.1 mg/ml sodium pyruvate, 100 U/ml penicillin and 100 µg/ml streptomycin. In both cases monocytes were differentiated into macrophages by culturing them in the presence of phorbol 12-myristate 13-acetate (PMA, Sigma Chemical, St. Louis, MO [25]) for 48 h.

Some cells were pre-treated with a p38-MAPK inhibitor (10 µM SB 202190, 24 h; Sigma Chemical, St. Louis, MO). In other experiments the following functional antibodies were employed: polyclonal antibody against CD36 (0.2 µg/µl, 3 h, Santa Cruz Biotechnology, CA, USA); monoclonal antibody against TSP-1 (0.2 µg/µl, 3 h; Santa Cruz Biotechnology), horseradish peroxidase-conjugated goat anti-mouse IgG (0.2 µg/µl; 3 h; Pierce, Rockford, IL USA); or goat anti-rabbit IgG (0.2 µg/µl; 3 h, Pierce).

Human peripheral blood mononuclear cells (PBMC) were isolated from healthy donors by Ficoll density gradient centrifugation. Monocytes were plated in 12-well tissue culture plates and matured to macrophages by culturing in X-Vivo 15 medium (BioWhittaker) supplemented with 1% human serum and 20 ng/nl recombinant human M-CSF (Peprotech, France) at 37°C in 5% CO_2_ for 6 days.

Hypoxia (3% O_2_) was established by incubating the cells for 5 h in a CO_2_/O_2_ incubator (model INVIVO_2_ 400, RUSKINN Technology Ltd, Pencoed, UK) with a blend of 5% CO_2_ and the desired percentage of O_2_ and N_2_ up to a total of 100%. Normoxic controls were obtained by incubating the cells at 21% O_2_.

#### RNA interference

U937 cells were transfected with a vector-targeting human HIF-1α (miHIF-1α) or a non-targeting control vector (mock), as described previously [26]. Lipofectamine-2000 (Invitrogen Life Technologies, Barcelona, Spain) was employed as a transfection reagent and used according to the manufacturer’s instructions. Twenty-four hours after transfection the cells were incubated for 5 h in normoxic or hypoxic conditions, as described above.

#### Neutrophil isolation, apoptosis and staining

Human peripheral blood polymorphonuclear (PMN) cells were isolated from whole blood of healthy volunteers using sodium citrate as an anticoagulant [27]. Samples were incubated with dextran (3%) for 45 min. PMN cells in the supernatant were separated by gradient density centrifugation (250 g, 25 min) with Ficoll-Paque™ Plus. Following red blood cell lysis, neutrophils were washed (HBSS without Ca^2+^ or Mg^2+^) and re-suspended in complete RPMI medium. To induce spontaneous apoptosis, neutrophils were cultured at 37°C for 24 h (5×10^6^ cells/ml in cultured medium without serum in a humidified atmosphere containing 5% CO_2_). Purity of isolation and apoptosis of neutrophils were assessed by Wright’s Giemsa staining. Neutrophil preparations with more than 90% apoptotic cells were labeled with 5-(and-6)-carboxy fluoresceindiacetatesuccinimidyl ester (CFSE) (Invitrogen Life Technologies, Barcelona, Spain) following the manufacturer’s instructions. Labelled cells were used as targets in the phagocytosis assay.

#### Phagocytosis assay

Following a 4 h-period of normoxic or hypoxic conditions, macrophages were co-cultured with CFSE-labeled apoptotic neutrophils at a phagocyte-to-target ratio of 1∶10. One hour later, cells were washed thoroughly with PBS. U937 cells were stained with Hoechst 33342 (Sigma-Aldrich, Steinheim, Germany) in order to visualize the nuclei and with a fluorescent mitochondrion-selective probe (MitoTracker® Red 580, Invitrogen Life Technologies, Barcelona, Spain) in order to define the cytoplasm area, and were then fixed with paraformaldehyde 4% for 10 min. Samples were analyzed with a fluorescent microscope (IX81, Olympus, Hamburg, Germany) and the CFSE fluorescent signal was quantified using the static cytometer software ‘Scan’ version 2.03.2 (Olympus, Hamburg, Germany). This system automatically counts the total number of cell nuclei per field and the number of phagocytic cells (green fluorescence on red fluorescence). All treatments were performed in duplicate in 12-well plates, and 20 images per well (around 2000 cells) were recorded. Results are expressed as intensity of fluorescence in arbitrary units.

#### Protein extraction and western blot analysis of HIF-1α, CD36 and TSP-1 expression

U937cells (2.5·10^6^ cells) were suspended and incubated on ice for 15 min with 50 µl of lysis buffer (10 mM HEPES, pH 7.5, 2 mM MgCl_2_, 1 mM EDTA, 1 mM EGTA, 10 mM NaCl, 1 mM DTT, 10 mM NaF, 0.1 mM Na_3_VO_4_, 0.2% NP40, 1 mM Pefabloc SC (AEBSF) (Roche Diagnostics GmbH, Mannheim, Germany), supplemented with Protease Inhibitor Cocktail (Roche Diagnostics GmbH, Mannheim, Germany). Lysates were centrifuged for 10 min at 4°C (16000 g). Supernatants were considered cytosolic extracts. Pellets were sonicated for 10 min in 50 µl nuclear extraction buffer (25 mM HEPES, pH7.5, 500 mM NaCl, 1 mM DTT, 10 mM NaF, 10% Glycerol, 0,2% NP40, 5 mM MgCl_2_, 1 mM Pefabloc SC (Roche Diagnostics GmbH, Mannheim, Germany) supplemented with Protease Inhibitor Cocktail (Roche Diagnostics GmbH, Mannheim, Germany). Nuclear lysates were centrifuged for 10 min at 4°C (16000 g). Supernatants were considered nuclear extracts. Total protein concentration in lysates was quantified using the Pierce BCA protein assay kit (Pierce, Rockford, IL USA). Equal amounts of protein were loaded onto SDS/PAGE gels and analyzed by Western blot, as described previously [10]. TSP-1 gels were run in non-reducing conditions. Membranes were blocked with 5% non-fat dry milk in TBS-T (20 mM Tris/HCl pH 7.2, 150 mM NaCl and 0.1% Tween 20) and incubated overnight with a monoclonal antibody against human HIF-1α (dilution 1∶250; BD Biosciences, San Jose, CA), human TSP-1 (dilution 1∶300; Thermo Scientific, Runcorn, Cheshire WA7 1PR, UK), human CD36 (1∶250; Abcam, Cambridge, UK) or Actin (dilution 1∶10000; Sigma-Aldrich, MO, USA). Protein bands were detected by LAS-3000 (Fujifilm) during incubation with horseradish peroxidase-conjugated goat anti-mouse IgG (dilution 1∶2500; Pierce, Rockford, IL USA) or goat anti-rabbit IgG (1∶5000; Pierce, Rockford, IL USA) following treatment with supersignal west picochemiluminescent substrate (Pierce, Rockford, IL USA). Protein expression was quantified by means of densitometry using Image Gauge Version 4.0 software (Fujifilm Global, Barcelona, Spain). Data were normalized to actin and results are expressed as fold induction vs control group.

### RNA Extraction and PCR Analysis

Total RNA was isolated from U937cells using the RNeasy Mini kit (Qiagen, Valencia, CA, USA). cDNA was synthesised and real-time PCR was performed as described previously [26]. Specific oligonucleotides for HIF-1α (5′-GAAAGCGCA AGTCCTCAAAG-3′ and 5′-TGGGTAGGAGATGGAGATGC-3′), TSP-1 (5′-AGAGAACAGAGCCCCAC AGA-3′ and 5′-CCCAAAATATCCTGGGAGGT-3′), and CD36 (5′-CAGTTGGAGACCTGCTTATCC-3′ and 5′-GCGTCC TGGGTTACATTTTC-3′) were designed according to reported sequences. Actin (5′-GGAC TTCGAGCAAGAGATGG-3′ and 5′-CTGTACGCCAACACAGTGCT-3`) expression was used as an internal control. The threshold cycle (C_T_) was determined and relative gene expression was expressed as follows: change in expression (fold) = 2^−Δ(ΔC^_T_^)^ where ΔC_T_ = C_T_ (target) - C_T_ (housekeeping), and Δ(ΔCT) = ΔC_T_ (treated) - ΔC_T_ (control).

### Static Cytometry

PBMC and U937-derived macrophages were exposed to hypoxia for 5 h. After treatment, cells were fixed with 2% paraformaldehyde, permeabilized with 0.1% Triton-X100, and then stained with polyclonal antibody against CD36 (1∶200, Santa Cruz Biotechnology, Santa Cruz, CA). FITC or TexRed labeled goat anti-rabbit IgG (H + L) (1∶200, Abcam, Cambridge, UK) were used as the secondary antibody, and 1 µM Hoechst 33342 (Sigma-Aldrich, Steinheim, Germany) was added to stain nuclei. Fluorescence (18 images per well) was visualized using the fluorescence microscope and the fluorescent signal was quantified using the static cytometer software ‘Scan’ version 2.03.2.

### Chromatin Immunoprecipitation

U937 cells were differentiated to macrophages. Following the hypoxia/normoxia treatment for 5 h, cells were washed twice with PBS and cross-linked with 1% formaldehyde at room temperature for 10 min. Cells then were rinsed with ice-cold PBS twice and collected into 100 mM Tris-HCl (pH 9.4), 10 mM DTT and incubated for 15 min at 30°C and centrifuged for 5 min at 2000 g. Cells were washed sequentially with 1 ml of ice-cold PBS, buffer I (0.25% Triton X-100, 10 mM EDTA, 0.5 mM EGTA, 10 mM HEPES, pH 6.5), and buffer II (200 mM NaCl, 1 mM EDTA, 0.5 mM EGTA, 10 mM HEPES, pH 6.5). Cells were then resuspended in 0.3 ml of lysis buffer (1% SDS, 10 mM EDTA, 50 mM Tris-HCl, pH 8.1, 1× protease inhibitor cocktail (Roche Diagnostics GmbH, Mannheim, Germany) and sonicated three times for 10 s each at the maximum setting (Branson, Digital Sonifier) followed by centrifugation for 10 min. Supernatants were collected and diluted in buffer (1% Triton X-100, 2 mM EDTA, 150 mM NaCl, 20 mM Tris-HCl, pH 8) followed by immunoclearing with 2 µg sheared salmon sperm DNA, 20µl pre-immune serum and protein A-sepharose (45 µl of 50% slurry in 10 mM Tris-HCl, pH 8, 1 mM EDTA) for 2 h at 4°C. Immunoprecipitation was performed overnight at 4°C with anti-HIF1α antibody (Novus Biologicals, CO, USA), or with control IgG antibody (Pierce). After immunoprecipitation, 45 µl protein A-Sepharose and 2 µg of salmon sperm DNA were added and the incubation continued for 1 h. Precipitates were washed sequentially for 10 min each in TSE I (0.1% SDS, 1% Triton X-100, 2 mM EDTA, 20 mM Tris-HCl, pH 8, 150 mM NaCl), TSE II (0.1% SDS, 1% Triton X-100, 2 mM EDTA, 20 mM Tris-HCl, pH 8, 500 mM NaCl), and buffer III (250 mMLiCl, 1% NP-40, 1% deoxycholate, 1 mM EDTA, 10 mM Tris-HCl, pH 8). Precipitates were then washed three times with TE buffer and extracted three times with 1% SDS, 100 mM NaHCO_3_. Eluates were pooled and heated at 65°C for at least 6 h to reverse the formaldehyde cross-linking. DNA fragments were purified with a Montage PCR Kit (Millipore, Germany). PCR was performed using PCR Master (Roche Diagnostics GmbH, Mannheim, Germany) with the following primers: 5′-AGATCTTTCGTTAAACCCCTGGTCCG-3′ and 5′-CTCGAGTCATGTCCTTTCTCACGTCCA-3′, detecting the region −33 to−493 in *TSP-1* promoter (35 cycles). The PCR products were separated by electrophoresis in 2% agarose gel.

### Electrophoretic Mobility Shift Assay

Nuclear extracts from *miHIF1α* or mock-transfected and non-transfected U937 cells were obtained as described above. Synthetic oligonucleotides (biotin-labelled) were synthesized (TIB MOLBIOL GmbH Berlin, Germany) and used as probes in electrophoretic mobility shift assays (EMSAs). Analysis revealed what appeared to be a hypoxic response element (HRE) motif (-62 TCA CAA TCT GGA CGT GAG AAA GGA CAT-35) in the *TSP-1* promoter. A mutated probe-binding site (TSP-1 mt: TCA CAA TCT GGA AAA AAG AAA GGA CAT) or excess unlabelled probes (XS) were used as controls. Nuclear extracts (10µg) were incubated for 5 min with or without an excess unlabelled probe in DNA binding buffer (Pierce) supplemented with 5 mM MgCl_2_, 50 ng/µl poly-d[I-C] (Pierce), 0.05% NP40 (Pierce) and 2.5% glycerol at room temperature. The labelled probe (25 fmol) was then added to the reaction mixture and incubated for 30 min at room temperature in a final volume of 20 µl. DNA-protein was resolved in a 6% non-denaturing polyacrylamide gel, as described previously. DNA-protein complexes were transblotted to Biodyne® B nylon membrane (Pierce, Rockford, IL USA), probed with streptavidin-horseradish peroxidase conjugate (Pierce, Rockford, IL USA) and developed by enhanced chemiluminescence.

### Immunohistochemical Studies

Patients clinically diagnosed with typical Crohn's disease and ulcerative colitis underwent a colonoscopy or sigmoidoscopy during which biopsy specimens were taken, from damaged and non-damaged mucosa (Table 1). HIF-1α, p38-MAPK and CD36 immunostaining was performed in representative 5 µm sections of paraffin-embedded tissues. Antigen retrieval was carried out with α-chymotrypsin for HIF-1α antibody (37°C, 20 min) or sodium citrate buffer pH = 9 for CD36 and p38-MAPK antibody (100°C, 20 min). In all cases, endogenous peroxidase activity was suppressed by immersion in 0.3% hydrogen peroxide (15 min). Following blocking with 5% horse serum, sections were incubated overnight (4°C) with a macrophage marker antibody (Vector Laboratories, Peterborough, UK) or a mouse monoclonal antibody against HIF-1α (Novus Biologicals, CO, USA, 1∶60), CD36 (1∶100) or p38-MAPK phosphorylated at Tyr182 (Novus Biologicals, CO, USA, 1∶200). A horse anti-mouse/rabbit biotinylated antibody (Vector Laboratories, Peterborough, UKr, 1∶200) was employed as a secondary antibody. The VECTASTAIN elite ABC system Kit (Vector Laboratories, Peterborough, UK) was used to develop. All tissues were counterstained with hematoxylin and the specificity of the immunostaining was confirmed by the absence of staining in analogous tissue sections when the primary or secondary antibodies were omitted. An area of 0.135 mm^2^ was analyzed for quantitative analysis.

**Table 1:** Patient clinical information.

	Ulcerative colitis	Crohn’s disease
**Number of patients**	10	4
**Age**		
17–40 years	3	1
>40 years	7	3
**Gender**		
Male	3	3
Female	7	1
**Location**		
Colonic	10	2
Ileum	–	2
**Extent of UC**		
Pancolitis	3	
Left-sided colitis	6	
Proctitis	1	
**Concomitant medication**		
Corticosteroids	**5**	2
5 ASA	9	–
AZA	1	3
Anti TNF	1	1

### Statistical Analysis

Data are expressed as mean ± s.e.m. and were compared by analysis of variance (one way-ANOVA) with a Newman-Keuls post hoc correction for multiple comparisons or a t-test when appropriate. A P value<0.05 was considered to be statistically significant. The correlation between CD36 and HIF-1 or CD36 and p38-MAPK was analyzed using the Spearman’s correlation coefficient.

## Results

### Hypoxia Increases the Phagocytosis of Apoptotic Neutrophils by Macrophages

**Figure 1:**
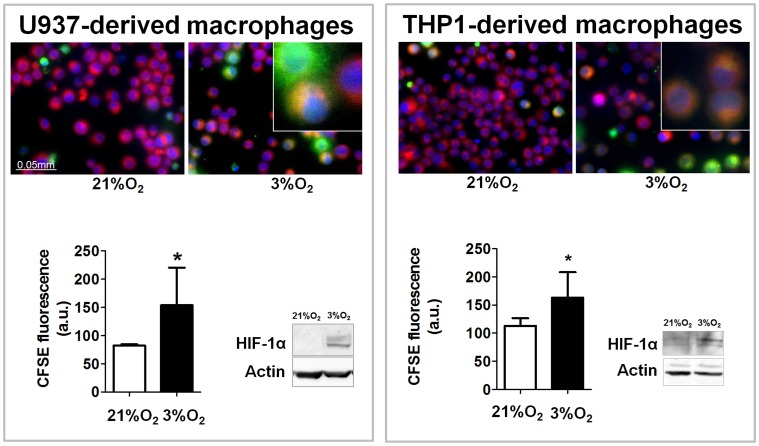
Hypoxia increases phagocytosis of apoptotic neutrophils by human macrophages. Representative images show phagocytosis of apoptotic neutrophils by U937 or THP1 cells in normoxia and hypoxia. In all cases blue staining identifies the nuclei, red staining defines the cytoplasm of the cells, and green staining indicates neutrophils. This system automatically counts the total number of cell nuclei per field and the number of cells exhibiting green fluorescence on red fluorescence (phagocytic cells). Inserts show magnification of the image. Results are expressed as intensity of fluorescence in arbitrary units. Bars in the graphs represent mean± SEM (*n>*3). Groups were compared using t-test analysis. Significant difference from the respective group in normoxic conditions is shown by *P<0.05. Western blot showing HIF-1α stabilization induced by hypoxia in U937 or THP1 cells.

Phagocytosis of CFSE-labelled apoptotic neutrophils was analyzed by static cytometry. As shown in Fig. 1, hypoxia enhanced the phagocytic activity (analyzed as intensity fluorescence in the assay) of both U937 and THP1 macrophages compared with normoxia. Western blotting revealed that incubation of U937 and THP1 macrophages in hypoxic conditions (3% O_2_) induced HIF-1α stabilization.

### Hypoxia Induces the Expression of CD36 and TSP-1 and HIF-1α Stabilization through a p38-MAPK-dependent Mechanism

**Figure 2:**
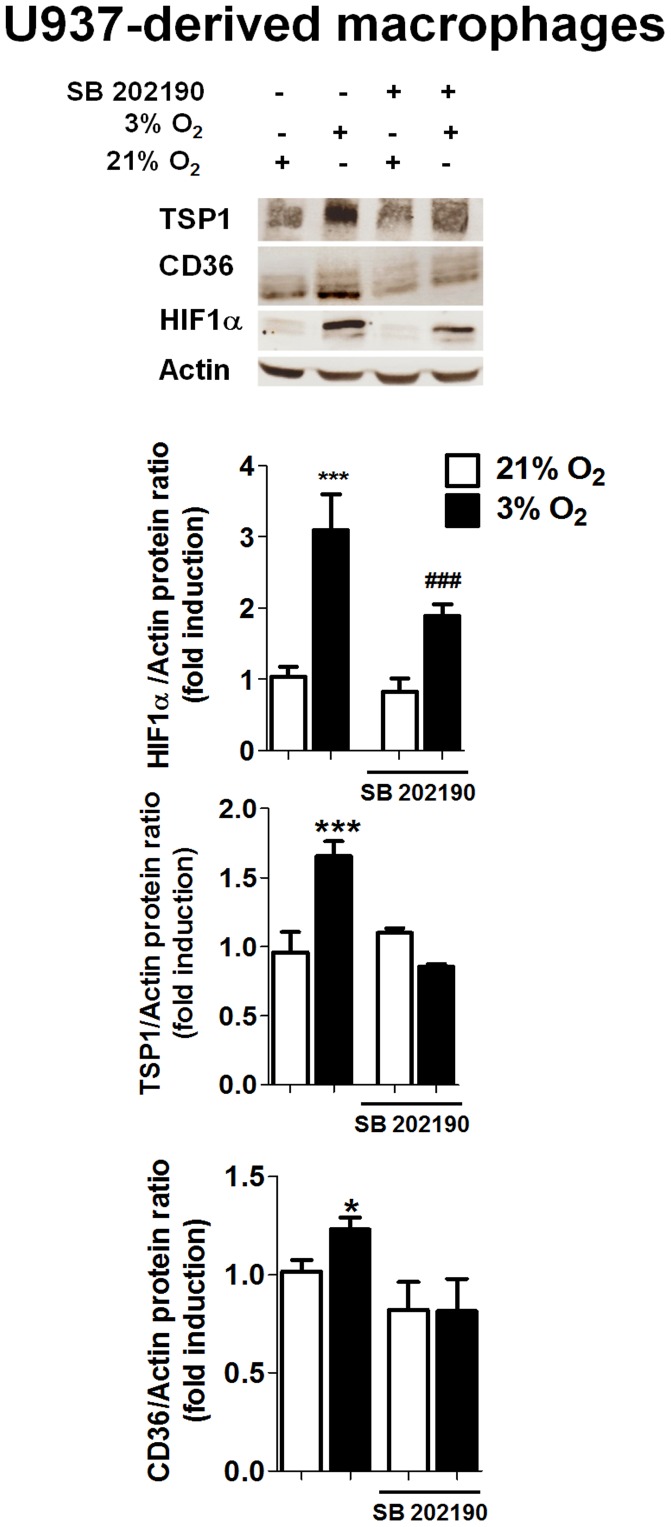
Hypoxia induces TSP-1 and CD36 expression and HIF-1α stabilization through activation of p38-MAPK. U937 cells were maintained under normoxia or hypoxia in the presence or absence of SB 202190 (a p38-MAPK inhibitor, 10 µM, 24 h) and levels of proteins were determined by Western blot. Graphs show quantification of HIF-1α, TSP-1 and CD36 by densitometry. In hypoxia, cells treated with SB 202190 exhibited significantly lower protein expression of HIF-1α, TSP-1 and CD36 than cells treated with vehicle. In all cases bars represent mean± SEM (*n>*3). Comparisons between groups were performed using ANOVA followed by a Newman Keuls test. *P<0.05 and ***P<0.001 with respect to all groups in the same graph and ^###^P<0.001 vs. bars in normoxia.

CD36 protein expression was detected in control U937cells (Fig. 2). Hypoxia induced a slight but significant increase in CD36 protein expression compared with normoxia as analyzed by western blot. This increase was confirmed by fluorescence static cytometry in both U937 cells and primary macrophages obtained from buffy coat (Figure S1). In a similar manner, TSP-1 protein expression was detected in control U937cells and hypoxia induced a significant increase in its expression compared with normoxia (Fig. 2).

The role of the p38-MAPK pathway in the effects of hypoxia on CD36 and TSP-1 expression and HIF-1α stabilization was studied by applying SB 202190, a p38-MAPK inhibitor. As shown in Fig. 2, treatment of cells with SB 202190 significantly decreased the protein expression of CD36 and TSP-1 induced by hypoxia, while it did not significantly modify levels of either protein in normoxia. This drug significantly undermined the stabilization of HIF-1α induced by hypoxia (Fig. 2).

### HIF-1 Mediates Phagocytosis and the Induction of CD36 and TSP-1 Induced by Hypoxia

**Figure 3:**
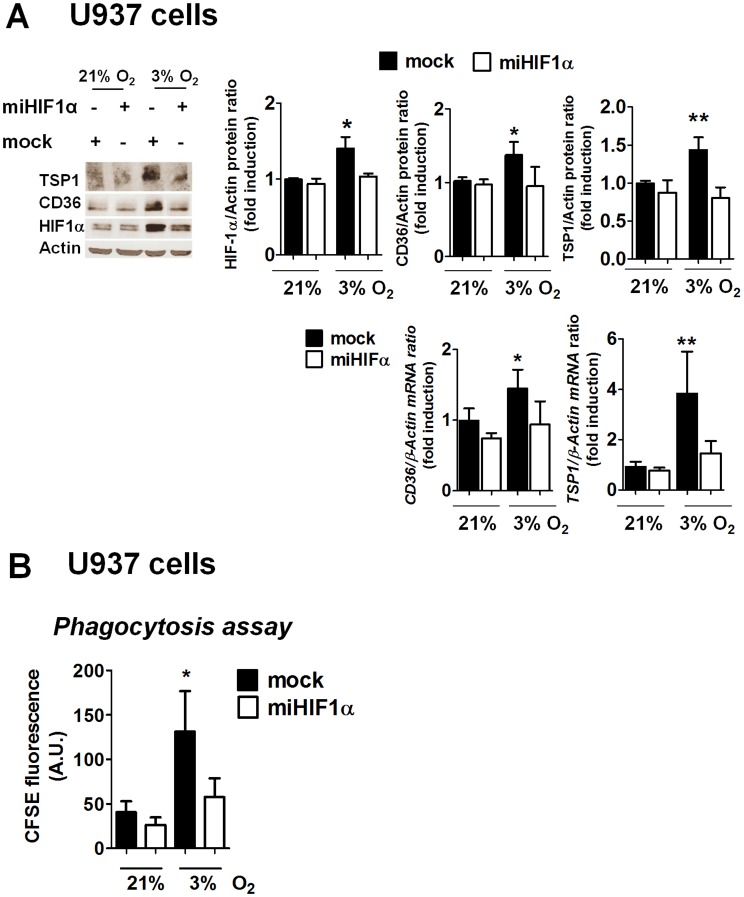
HIF-1 mediates phagocytosis and the increased expression of TSP-1 and CD36 induced by hypoxia. A) Western blot showing HIF-1α stabilization, CD36 and TSP-1 in U937 cells transfected with *miHIF-1α* and mock-transfected cells (controls). Graphs show quantification of the HIF-1α, TSP-1 and CD36 proteins by densitometry and mRNA expression of *CD36* and *TSP-1* by RT-PCR in mock-transfected cells and cells treated with *miHIF-1α*. B) Graph shows phagocytosis of apoptotic neutrophils in *miHIF-1α* or mock-transfected cells. Bars in the graphs represent mean± SEM (*n>*3). Comparisons between groups were performed using ANOVA followed by a Newman Keuls test. *P<0.05 or **P<0.01 with respect to all bars in the same graph.

Expression of HIF-1α in U937 macrophages was knocked down with miRNA as previously described [26]. HIF-1α protein levels in hypoxia were significantly lower in cells expressing *miHIF-1α* than in mock cells (Fig. 3A). *CD36* and *TSP-1* mRNA expression was detected in control U937cells in normoxia and it was increased by hypoxia (Fig. 3A). The hypoxia-induced increase in the expression of CD36 and TSP-1 proteins and mRNA was abolished in cells treated with *miHIF-1α,* thus confirming the involvement of HIF-1 in the up-regulation of these genes during hypoxia. Phagocytosis of CFSE-labelled apoptotic neutrophils was analyzed in *miHIF-1α* and mock macrophages. As shown in Fig. 3B, hypoxia enhanced the phagocytic activity of mock macrophages compared with normoxia, but it failed to do so in *miHIF-1α* cells.

### HIF-1 Binds to the Promoter Region of TSP-1

**Figure 4:**
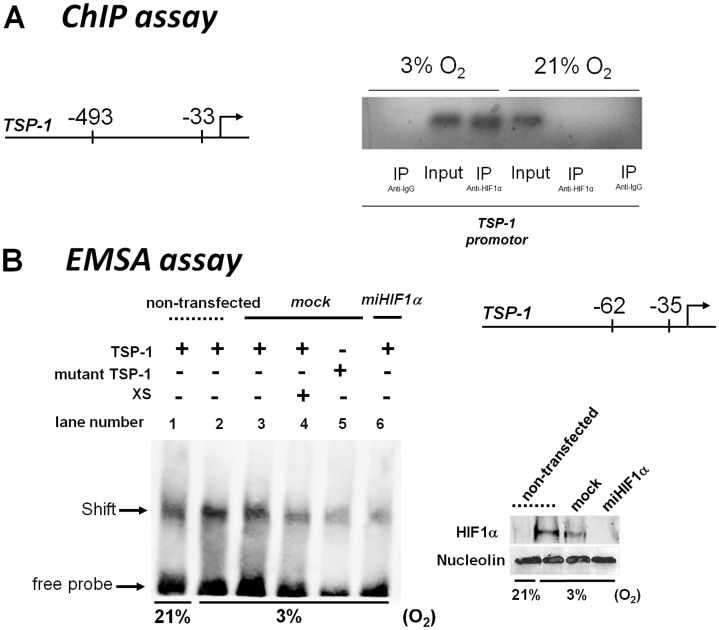
Recruitment of HIF-1 to the promoter of *TSP-1* gene. A) Results show a representative chromatin immunoprecipitation (ChIP) experiment performed in samples from U937-derived macrophages in normoxia or hypoxia. Chromatin was immunoprecipitated with anti-HIF-1α antibody, or a non-related antibody anti-IgG as a control. An aliquot of the input chromatin is also shown. Primers specific to the promoter region for TSP-1 gene were used to amplify the DNA isolated from the ChIP assay. B) HIF-1α expression in nuclear lysates derived from non-transfected cells and from *miHIF1α* or mock-transfected U937cells exposed to normoxia or hypoxia. Interactions between HIF-1α and HRE of the TSP-1 promoter gene were examined by EMSA using synthetic oligonucleotides and nuclear lysates derived from transfected or non-transfected cells exposed to normoxia or hypoxia. Specificity was determined with excess unlabelled probed (XS) or mutated probe (n = 3).

Analysis of the TSP-1 gene promoter identified some HIF-1 binding sites (HRE sequence) between positions −493 and −33 relative to the transcription starting site. To examine the potential role of HIF-1α on the expression of TSP-1, ChIP assays were performed with an affinity-purified antibody directed against HIF-1α (Fig. 4A). DNA was extracted from the input, bound (anti-HIF1α), and unrelated (anti-IgG) antibody fractions; equal amounts from each fraction were amplified using primers specific for the TSP-1 promoter region. The binding was determined by the relative intensity of ethidium bromide fluorescence compared with the input control. Our data show HIF-1α binding to the TSP-1 gene in hypoxia. Afterwards we employed EMSA to examine the binding of HIF-1 to the HRE consensus between positions −62 and −35 relative to the transcription starting site, in the TSP-1 promoter. Hypoxia increased the binding of HIF-1 to its consensus sequence in the TSP-1 promoter in both non-transfected and control mock-transfected cells (Fig. 4B). The specificity of the assay was confirmed by the fact that binding was diminished when cells were transfected with *miHIF1α* (lane 6), when a mutated promoter was used (lane 5) or with an excess of unlabelled probe (XS, lane 4). Collectively, these results indicate that hypoxia elicits nuclear accumulation of HIF-1α, which binds to the consensus HRE located within the TSP-1 promoter.

### CD36 and TSP-1 Mediate Phagocytosis Induced by Hypoxia

**Figure 5:**
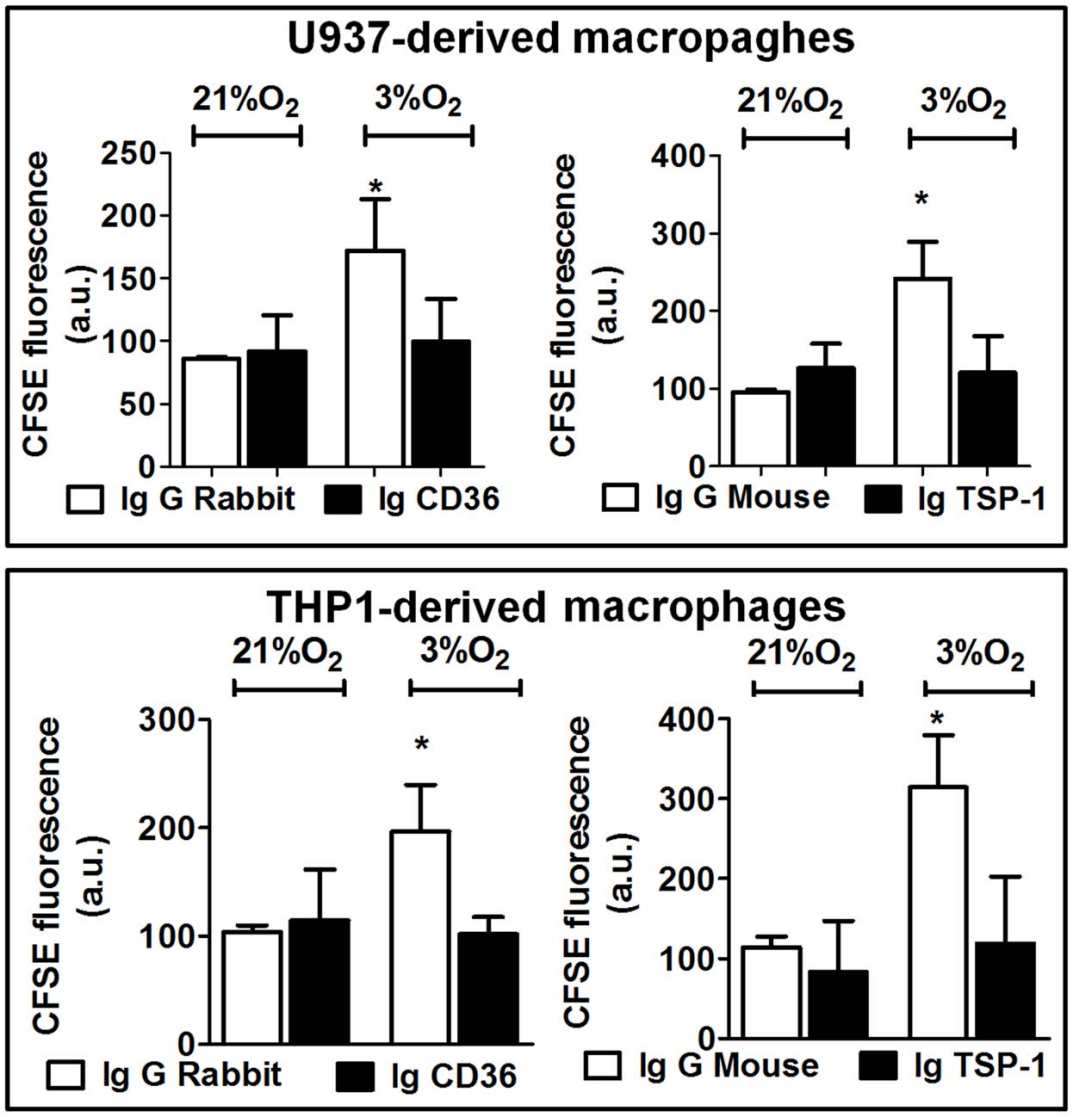
Role of CD36 and TSP-1 in phagocytosis mediated by macrophages. Graphs show the effects of CD36 and TSP-1 functional antibodies or control IgG on phagocytosis of apoptotic neutrophils mediated by U937 cells or THP1 cells. In both cases, blockade of CD36 or TSP-1 significantly reduced hypoxia-induced phagocytosis. Data show the intensity of fluorescence in arbitrary units (quantified by static cytometry). Bars represent mean± SEM (*n>*3). Groups were compared using ANOVA followed by a Newman Keuls test. *P<0.05 shows significant difference with respect to all groups in the same graph.

Specific functional antibodies were employed to block the activity of CD36 and TSP-1 in U937 and THP1 cells and thus evaluate the role of these molecules in phagocytosis. While hypoxia induced a significant increase in phagocytosis in IgG control cells, it failed to do so in cells treated with a monoclonal antibody against CD36. This antibody did not significantly modify phagocytosis in normoxia (Fig. 5). In a similar manner, a TSP-1 antibody significantly reduced the increase in phagocytosis induced by hypoxia (Fig. 5). In neither case did functional blockade of TSP-1 significantly modify phagocytosis in normoxic conditions.

### CD36 Expression Correlates with HIF-1 and p38-MAPK Expression in the Damaged Mucosa of Patients with IBD

**Figure 6:**
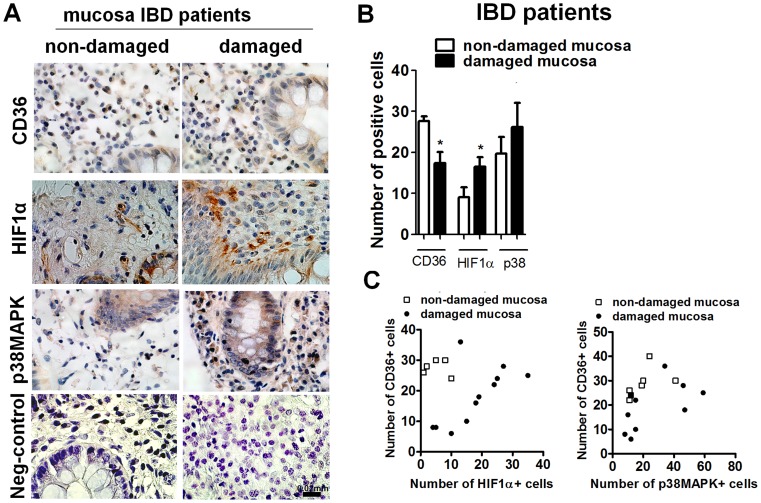
HIF-1, p38-MAPK and CD36 correlates in the inflamed mucosa of patients with inflammatory bowel disease. A) Representative microphotographs showing HIF-1α, p38-MAPK and CD36 immunostaining in the damaged and non-damaged mucosa of patients with inflammatory bowel disease. Biopsy specimens of the intestine were excised, formalin-fixed, paraffin-embedded, cut into 5 µm slices, and stained with hematoxylin; B) Graph shows a quantitative analysis of the number of HIF-1α, p38-MAPK or CD36 positive cells in a total area of 0.135 mm^2^ of the mucosa of patients with IBD. Bars in the graph represent mean± SEM (*n>*3). Significant difference from the respective non-damaged mucosa is shown by *P<0.05. C) Graphs show a positive and significant correlation between CD36 and HIF-1α (R Spearman = 0.7170, P = 0.0087**, n = 12) and p38-MAPK and CD36 (R Spearman = 0.6525, P = 0.0215*, n = 12) immunostaining at the damaged mucosa of patients with IBD. No correlation was observed between CD36 and HIF-1α (R Spearman = −0.0513, P = 0.95, n = 5), or p38-MAPK and CD36 (R Spearman = 0.5204, P = 0.2311, n = 7) immunostaining at the non-damaged mucosa.

In order to analyze the relevance of CD36 expression by HIF-1 in inflammation, we performed immunohistochemical studies of the damaged and non-damaged mucosa of patients with inflammatory bowel disease. As can be seen in Fig. 6A, cells of the lamina propria of the non-damaged mucosa, morphologically identified as macrophages, exhibited CD36 expression. The number of CD36-positive cells was significantly lower in the damaged mucosa than in non-damaged mucosa (Fig. 6B). The analysis of HIF-1α stabilization revealed a very low expression of this transcription factor in the lamina propria of non-damaged mucosa and an increased expression in the damaged mucosa (Fig. 6A, B). Evaluation of p38-MAPK immunostaining showed that this enzyme was widely expressed in non-damaged mucosa and the signal was increased in damaged mucosa (Fig. 6A, B).

A detailed analysis of the immunostaining in the damaged mucosa of patients with IBD showed a positive and significant correlation between HIF-1α and CD36-positive cells (R Spearman = 0.7170, P = 0.0087**, n = 12). In contrast, no significant correlation was observed between CD36 and HIF-1α immunostaining in non-damaged mucosa (R Spearman = −0.0513, P = 0.95, n = 5) (Fig. 6C). Interestingly, a significant correlation was also observed in the damaged mucosa between p38-MAPK and CD36 (R Spearman = 0.6525, P = 0.0215*, n = 12) while no significant correlation was observed in the non-damaged mucosa (R Spearman = 0.5204, P = 0.2311, n = 7) (Fig. 6C).

## Discussion

The findings of the present study demonstrate that HIF-1 transcriptional regulation plays an important role in hypoxia-induced phagocytosis of apoptotic neutrophils mediated by macrophages.

Phagocytosis by macrophages is critical for the uptake and degradation of infectious agents and senescent cells, a process implicated in development, tissue remodeling, the immune response and inflammation [28]. The present results show that exposure of human macrophages to hypoxia leads to an increase in the rate of phagocytosis of apoptotic neutrophils. Previous studies have shown an increase in bacterial phagocytosis by murine macrophages in hypoxia [15], [16], [29] and we have observed a similar process in *E coli* phagocytosis by human macrophages (data not shown). Considered together, the evidence points to the existence of a general mechanism that is activated in macrophages by hypoxia and which leads to an increase in phagocytic activity irrespective of the particle that is to be recognized and internalized. By highlighting the induction of neutrophil phagocytosis by low oxygen levels, our data extend the pathophysiological relevance of hypoxia from the initial stages of the inflammatory process to the resolution of inflammation.

CD36 in macrophages acts as a class B scavenger receptor known to recognize, bind with and internalize apoptotic neutrophils [19], [20]. CD36 regulation by hypoxia has been studied and contradictory results have been reported [13], [30]. The present study, by using different experimental approaches, demonstrates a slight but significant increase in CD36 expression induced by hypoxia. In addition we also show an increased up-regulation of TSP-1 expression by hypoxia in macrophages. It has been report that CD36 binds to TSP-1 as a pattern recognition receptor, thus constituting a phagocytically active ternary complex which mediates the phagocytosis of neutrophils [21]. Hypoxia has been implicated in the activation of p38-MAPK [31], [32], and our present data reveal a role for this pathway in the hypoxia-induced expression of CD36 and TSP-1, since pharmacological blockade of the activity of these enzymes by SB 202190 significantly decreased their levels. The p38-MAPK signalling pathway is known to modulate the activity of HIF-1 [4], [5] and HIF-1 has been related to CD36 expression in endothelial vascular and smooth muscle cells [24]. Interestingly, the present study shows that inhibition of p38-MAPK significantly undermines the HIF-1α stabilization induced by hypoxia in macrophages, which suggests a role for HIF-1 in said expression. In line with this, our results demonstrate that the transcriptional activity of HIF-1 is involved in the effects of hypoxia on both CD36 and TSP-1 expression, since down-regulation of this transcription factor by transient transfection significantly decreased the mRNA and protein expression of both molecules. As far as we know, this is the first report of hypoxia-induced HIF-1-dependent generation of TSP-1 in macrophages. In addition, our results take current evidence one step further by confirming the binding of HIF-1 to an HRE sequence in the promoter region of the TSP-1 gene, which suggests that this gene is a direct target of HIF-1. In light of a previous study reporting CD36 as a target gene of HIF-1 [24], the present findings indicate that, in hypoxic macrophages, HIF-1 synchronizes the transcriptional up-regulation of these two genes, both of which are crucial to the process of phagocytosis.

A previous study by our group showed a correlation between the expression of HIF-1 in macrophages and the clearance of infiltrated neutrophils in the mesentery of aspirin-treated rats [33] which lead us to suggest the involvement of this transcription factor in phagocytosis of neutrophils. The present study by the selective diminution of HIF-1α in cultured macrophages demonstrates a role for HIF-1 in phagocytosis of apoptotic neutrophils. In addition we have evaluated the relevance of CD36 and TSP-1 up-regulation by hypoxia in the phagocytic activity of macrophages using function-specific antibodies. Results show that CD36 is required for the induction of macrophage-mediated phagocytosis of apoptotic neutrophils during hypoxia. In a similar manner, immunological blockade of TSP-1 abolished the increase in phagocytosis of apoptotic neutrophils induced by hypoxia. This is in accordance with a putative role for TSP-1 as a bridge between CD36 and membrane phospholipids of apoptotic cells [21]. Since CD36 and TSP-1 need each other to recognize and phagocyte apoptotic neutrophils, our results reveal that HIF-1 functions by promoting an effector response by which apoptotic cells are removed from hypoxic microenvironments. Regulation of these two genes by HIF-1 could be part of a wider response in which a subset of genes helps to resolve inflammation.

Finally, we have analyzed the pathophysiological relevance of CD36 regulation by HIF-1 and p38-MAPK in the intestinal mucosa of patients with inflammatory bowel disease. Our results show low HIF-1α stabilization and high CD36 expression in the non-damaged mucosa which leads us to suggest that transcription factors other than HIF-1 are involved in the expression of this scavenger receptor at the healthy mucosa. In this line constitutive CD36 expression has been shown to be regulated by several nuclear receptors, including PPARγ [22]. Interestingly gene expression of PPAR-γ is down-regulated in the damaged mucosa of patients with ulcerative colitis [34] which is in accordance with results in the present study showing a decrease in CD36 expression in damaged mucosa compared with non-damaged. In contrast to that observed in the non-damaged mucosa, a detailed analysis of the damaged mucosa revealed a positive and significant correlation between CD36 and HIF-1α immunostaining. Considering that HIF-1 is significantly increased in the damaged mucosa our results propose that at sites of inflammation where the mechanisms that modulate the constitutive expression of CD36 are down-regulated, the expression of this scavenger receptor may depend on HIF-1 activity. It is important to note that both down-regulation of PPAR-γ [35] and HIF-1α stabilization have been related to hypoxia. Taking into account that low oxygen levels are associated with inflamed tissue results lead us to suggest hypoxia as an important regulator of CD36 expression at sites of inflammation. Reinforcing this observation, both hypoxia and IBD have been related to p38-MAPK activity [4], [5], [36]–[38] and the present study shows high expression of p38-MAPK in the damaged mucosa of IBD patients. Positive immunostaining has been observed in epithelial cells and different cells of the lamina propria, including macrophages. Despite the high expression, the quantitative analysis of p38-MAPK immunostaining in cells of the lamina propria morphologically identified as macrophages shows a positive and significant correlation with CD36 expression. Taken together results suggest that CD36 expression at the damaged mucosa of IBD patients may depend on both p38MAPK and HIF-1 activity. It is important to note that in some samples we found a higher number of p38-MAPK positive cells than CD36 positive cells which suggests that some of the p38-MAPK positive cells are not expressing CD36. Considering that these cells have been identified as macrophages it seems likely that in the damaged mucosa of IBD patients, macrophages with a different pattern of expression may be present [39]. Further experiments are necessary to address this question.

In summary, our findings reveal a mechanism by which the transcriptional activity of HIF-1 coordinates induction of CD36 and TSP-1 expression in macrophages, a mechanism that mediates the enhanced phagocytosis of apoptotic neutrophils in hypoxia. In addition to its known pro-inflammatory action, HIF-1 may also constitute an important regulator in the resolution of inflammation.

## Supporting Information

Figure S1 **Hypoxia increases CD36 expression in PBMC and U937-derived macrophages.** Graphs show the effect of hypoxia on the expression of CD36 in PBMC and U937-derived macrophages. Results are expressed as intensity of fluorescence in arbitrary units. Bars in the graphs represent mean± SEM (*n = *3). Groups were compared using t-test analysis. Significant difference from the respective group in normoxic conditions is shown by *P<0.05.
(TIF)
