# Supplementary figures and images for: Induction of CD36 and Thrombospondin-1 in Macrophages by Hypoxia-Inducible Factor 1 and Its Relevance in the Inflammatory Process
Source: PLoS One. 2012 Oct 31;7(10):e48535. doi: 10.1371/journal.pone.0048535 (PMC3485304; doi:10.1371/journal.pone.0048535)

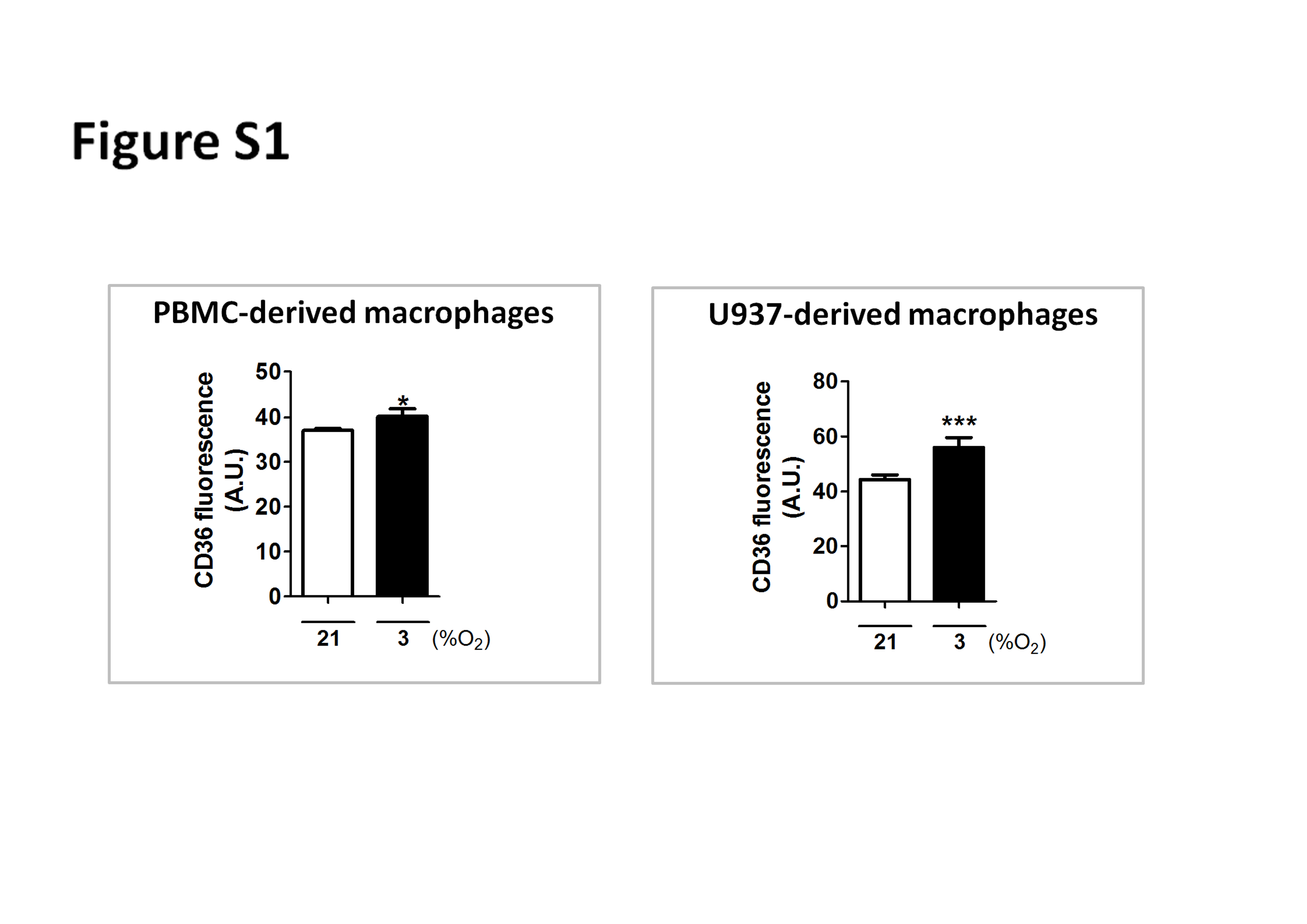

Supplement: Figure S1 — Hypoxia increases CD36 expression in PBMC and U937-derived macrophages. Graphs show the effect of hypoxia on the expression of CD36 in PBMC and U937-derived macrophages. Results are expressed as intensity of fluorescence in arbitrary units. Bars in the graphs represent mean± SEM (n = 3). Groups were compared using t-test analysis. Significant difference from the respective group in normoxic conditions is shown by *P<0.05. (TIF) [file pone.0048535.s001.tif]
